# miR-133b suppresses metastasis by targeting HOXA9 in human colorectal cancer

**DOI:** 10.18632/oncotarget.19212

**Published:** 2017-07-12

**Authors:** Xiao Wang, Juyuan Bu, Xingwei Liu, Wenfeng Wang, Weihua Mai, Baojun Lv, Jinlin Zou, Xiangqiong Mo, Xiaoling Li, Jingyu Wang, Bin Niu, Yunping Fan, Bingzong Hou

**Affiliations:** ^1^ Departments of General Surgery, The Fifth Affiliated Hospital of Sun Yat-Sen University, Zhuhai, Guangdong Province, China; ^2^ Departments of Preventive Medicine, The Fifth Affiliated Hospital of Sun Yat-Sen University, Zhuhai, Guangdong Province, China; ^3^ Departments of ENT - Head and Neck Surgery, The Fifth Affiliated Hospital of Sun Yat-Sen University, Zhuhai, Guangdong Province, China

**Keywords:** colorectal cancer, miR-133b, HOXA9, metastasis

## Abstract

Functions and mechanisms of microRNA (miRNA) involved in colorectal cancer (CRC) metastasis are largely unknown. Here, a miRNA microarray analysis was performed in CRC primary tissues and metastatic hepatic tissues to disclose crucial miRNA involved in CRC metastasis. MiR-133b was decreased and negatively correlated with metastasis in CRC. Overexpression of miR-133b significantly suppressed metastasis of CRC *in vitro* and *in vivo*. HOXA9 was identified as a direct and functional target of miR-133b. In addition, HOXA9 was negatively correlated with miR-133b and promoted CRC malignant progress. Moreover, miR-133b decreased HOXA9 expression, and subsequently downregulated ZEB1 and upregulated E-cadherin expression. Intriguingly, lower miR-133b and higher HOXA9 expression significantly contributed to poorer outcomes in CRC patients. Multivariate analysis indicated that miR-133b was an independent and significant predictor of CRC patient overall survival. In conclusion, we newly determined that miR-133b targeted the HOXA9/ZEB1 pathway to promote tumor metastasis in CRC cells. This axis provided insights into the mechanism underlying miRNA regulation of CRC metastasis and a novel therapeutic target for CRC treatment.

## INTRODUCTION

Colorectal cancer (CRC) is ordered as the second most prevalent cancer in women and the third most common cancer in men according to world health Organization [1]. Despite advances in therapeutic strategies, the clinical outcome and prognosis of CRC remains poor [2]. Tumor metastasis is a major cause of mortality in CRC [3]. Although many cell growth and metastasis-related genes were found, the molecular mechanisms that suppress tumor cell growth, migration and invasion are largely unknown [4–6]. Recently, there has been an attention on the characterization of microRNAs in oncogenesis and tumor suppressing. These microRNAs have been described to take part in a diversity of cancers, especially in cell malignant progression [7–10]. As such, microRNAs serve as important regulators of tumor development.

MiRNAs are endogenous non-coding RNAs conding∼22 nucleotides (nt) that can negatively regulate protein expression by inducing the degradation of target mRNAs or impairing their translation or both by specifically binding to the 3′-untranslated regions (3′-UTRs) of target mRNA. Accumulating data showed that miRNA may function as tumor suppressors or oncogenes [11]. Many miRNAs have been proved to play critical roles in human CRC, including miR-149, miR-150, miR-200 and etc. [12–17]. However, the exact CRC metastatic miRNAs had seldom been found.

In this study, we performed miRNA arrays in primary CRC tissues and metastatic hepatic sites to excavate miRNAs that drive CRC invasion and metastasis. MiRNAs that are differentially expressed in the metastatic sites may directly promote or restrain CRC metastasis. Among these differentially expressed miRNAs, miR-133b was often downregulated in CRC. Importantly, miR-133b was negatively associated with metastasis and poor prognosis and could be an independent indicator for CRC patient outcome. We showed that miR-133b over-expression inhibited proliferation, migration and invasion *in vitro* and *in vivo* by binding to HOXA9 3′ UTR in CRC cells. Our findings showed that miR-133b/HOXA9 axis is an important regulator in the development and progression of CRC and may be a candidate target for CRC treatment.

## RESULTS

### MiR-133b is lower expressed in CRC tissues

**Figure 1:**
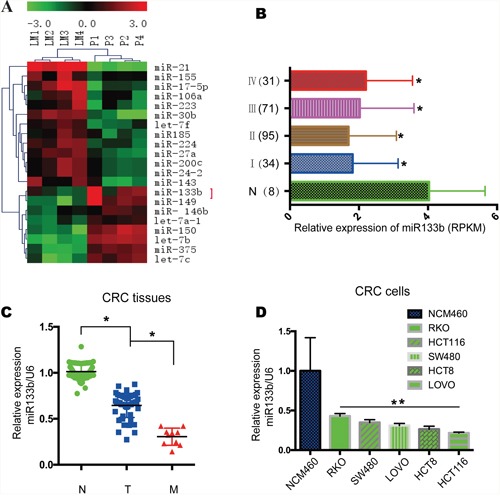
MiR-133b is downregulated in CRC tumor samples and cell lines **(A)** Microarray analysis of miRNA expression in CRC tissues from CRC primary cites (P) and hepatic metastatic sites of CRC (LM). **(B)** Relative expression of miR-133b in TCGA data, which contained 8 normal samples, 34 stage I, 95 stage II, 71 stage III and 31 stage IV samples. **(C)** qPCR analysis of miR-133b expression in 66 adjacent normal control tissues (N), 56 CRC without metastasis (T), and 10 CRC with metastasis (M). **(D)** qPCR analysis of miR-133b expression in normal cell lines and different CRC tumor cell lines.

To determine the roles of miRNAs in CRC metastasis, miRNA microarray analysis was conducted with four paired human primary CRC sample and metastatic hepatic tissue (Figure 1A). About all of the human miRNAs analyzed, a sum of 21 miRNAs were differentially expressed in the metastatic hepatic sample with at least 3-fold changes compared to the primary colon tumor sites. Among those differentially expressed miRNAs, miR-133b performed a highly important role in cancer, which is downregulated in CRC. To examine the expression of miR-133b in CRC, TCGA data was analyzed, which showed that miR-133b was significantly downregulated in CRC tissues compared to adjacent normal tissues (Figure 1B). Furthermore, 66 pairs of CRC primary tumor tissues and adjacent normal tissues were analyzed by qPCR. The results revealed that miR-133b expression was significantly lower in CRC tumor tissues compared to adjacent normal tissues (Figure 1C). A decrease of miR-133b in the 10 CRC tissues with metastasis was also remarked compared to primary tumors without metastasis (Figure 1C), which proposed that the down-regulation of miR-133b was strictly related to the progression of CRC metastasis. We further examined miR-133b expression in a group of human CRC cell lines and a normal cell line. Like the data collected from the CRC clinical samples, miR-133b was lower expressed in all of the CRC cell lines compared to the normal cell line (Figure 1D). Interestingly, The HCT116 and HCT8 cells, which possess high metastatic capacities, expressed the lowest levels of miR-133b. These results hinted that miR-133b is lower expressed in CRC and might inhibit CRC progression.

### MiR-133b suppresses CRC tumor proliferation and migration *in vitro*

**Figure 2:**
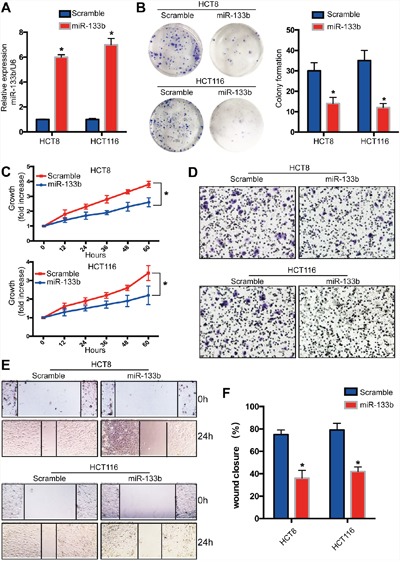
Recovery miR-133b expression in CRC tumor cells inhibits cell proliferation and migration *in vitro* MiR-133b was overexpressed in CRC cell lines by using miR-133b mimic (scramble or miR-133b). **(A)** qPCR analysis of miR-133b levels in CRC cell lines after transfection. **(B)** Cell colony formation assay. **(C)** Cell growth was analyzed at different time points after transfection. **(D)** Cell invasion was analyzed by way of gel invasion assay after virus infection. **(E** and **F)**. wound closure assay was detected after transfection. *P<0.05 versus scramble control.

To discover whether miR-133b inhibit the malignant phenotype of CRC cells, miR-133b mimic was transfected into HCT116 and HCT8 colon cancer cell lines to overexpress miR-133b (Figure 2A). The overexpression of miR-133b significantly repressed the proliferation of CRC cells (Figure 2B, 2C). These data showed that miR-133b might be a tumor suppressor and that its over-expression in cancer might inhibit cell proliferation. Given that the expression of miR-133b is negatively correlated with the metastatic feature of CRC, we wondered whether miR-133b suppressed CRC cell migration and metastasis. Transwell assays implied that miR-133b dramatically suppressed the migration of HCT116 and HCT8 cells when compared to the control groups (Figure 2D). Interestingly, the wound-healing assay revealed that miR-133b-treated CRC cell lines reduced wound closure compared to the control group (Figure 2E, 2F). In summary, restoring miR-133b could inhibit the proliferation and migration of CRC cells *in vitro*.

### MiR-133b Suppresses CRC Development and Metastasis *In Vivo*

**Figure 3:**
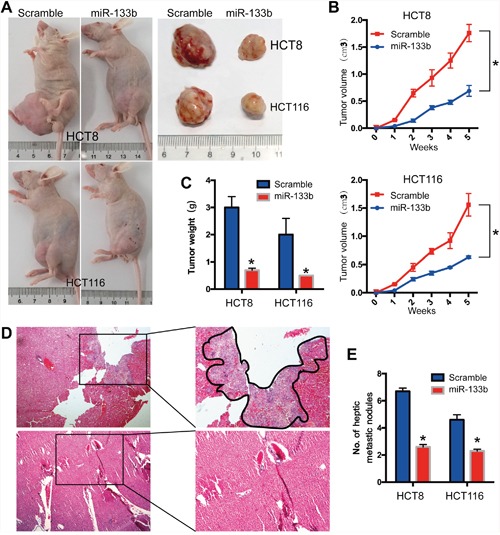
MiR-133b suppresses CRC tumorigenesis and metastasis *in vivo* HCT8 and HCT116 cells were transfected with miR-133b-mimic or scramble and then subcutaneously injected into nude mice. **(A)** *in vivo* subcutaneous tumor cell transplantation, the gross morphology of tumors are represented. **(B, C)** The statistical data for tumor volume **(B)** and tumor weight **(C)** are shown. **(D)** Representative image of hepatic metastatic sites. **(E)** Haptic metastatic tumor volume are shown. *P < 0.05, versus scramble control.

To further investigate the role of miR-133b in CRC development and metastasis *in vivo*, HCT8/HCT116-miR-133b cells or control cells were injected into nude mice through the subcutaneous injection. The tumor growth rate in the miR-133b group was significantly slower compared to control group in both the HCT8 and HCT116 transplanted xenograft tumor models (Figure 3A, 3B). At the fifth week after injection, the tumor volume and weight in the miR-133b group were significantly reduced compared to the control group (Figure 3B, 3C). To further determine the effect of miR-133b on metastasis *in vivo*, cells were inoculated via the portal circulation to assess metastatic activity upon hematogenous arrival in the liver. After six weeks, the mice were euthanized and the livers were fixed and examined. The numbers of hepatic metastatic nodules were significantly lower in the miR-133b group than were those in the scramble group (Figure 3D, 3E). In summary, these investigations showed that miR-133b significantly restrains CRC cell migration and invasion *in vitro* and *in vivo*.

### MiR-133b directly targets HOXA9 in CRC cells

**Figure 4:**
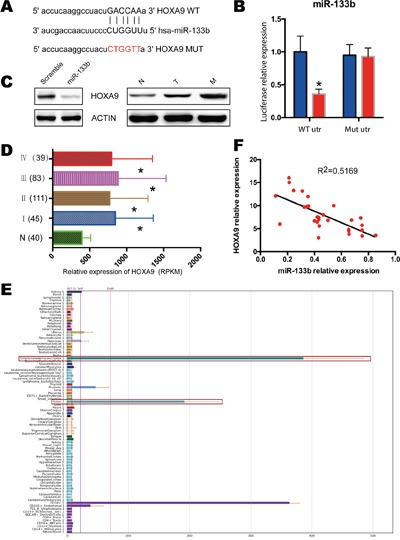
HOXA9 was identified as miR-133b targets in CRC **(A)** miR-133b targeting genes and the corresponding mutations. **(B)** examination of luciferase activity. Co-transfection of a wildtype or a mutant HOXA9 3′UTR with miR-133b mimics into HEK 293 cells. Firefly luciferase activity was measured and standardized by Renilla luciferase activity. **(C)** Left, HCT8 cells were infected with miR-133b mimics or scramble control, and protein levels of HOXA9 was analyzed with western blot. Right, the expression of HOXA9 in normal control (N), CRC without metastasis (T) and CRC with metastasis(M). 3 pair samples were used and a representative sample is shown. **(D)** Relative expression of HOXA9 in TCGA of CRC, which contained 40 normal samples, 45 stage I, 111 stage II, 83 stage III and 39 stage IV samples. **(E)** Screenshot of the profiles of the human gene HOXA9 within the BioGPS online portal. All data used for this study are available through the BioGPS database (biogps.org). **(F)** the expression correlation analysis for miR-133b and HOXA9 in 66 CRC tumor samples. *P < 0.05 versus scramble or healthy controls.

To refine the molecular mechanisms by which miR-133b suppresses CRC development and metastasis, we searched for potential target genes of miR-133b that might be implicated in the pathogenesis of CRC. The bioinformatics algorithm TagetScan was applied to predict HOXA9 as a putative target for miR-133b, which might take part in the progress of in CRC (Figure 4A). To validate the prediction, luciferase reporter constructs taking the 3′UTR miR-133b potential binding site or mutant binding sites of HOXA9 were constructed and co-transfected with miR-133b or a vector into HEK-293T cells. The luciferase assays unveiled that the overexpression of miR-133b significantly decreased the luciferase reporter, which showed that miR-133b interfered the expression of HOXA9 by binding the 3′ UTR of HOXA9 (Figure 4B). Moreover, the overexpression of miR-133b led to a significant reduction in HOXA9 expression (Figure 4C). We then identified the expression level of HOXA9 in CRC tissues and adjacent non-tumor mucosal tissues. Compared to the adjacent non-tumor mucosal tissues, the HOXA9 expressed significantly higher in primary CRC tissues and highest in the CRC tissues with metastasis (Figure 4C). Besides, TCGA data and BioGPS Gene Expression Atlas also confirmed the overexpression of HOXA9 in CRC tissues (Figure 4D, 4E). qPCR analysis of our 66 CRC samples showed that overexpression of HOXA9 was greatly correlated with the downregulation of miR-133b (Figure 4F).

### HOXA9 promoted CRC cell migration and invasion

**Figure 5:**
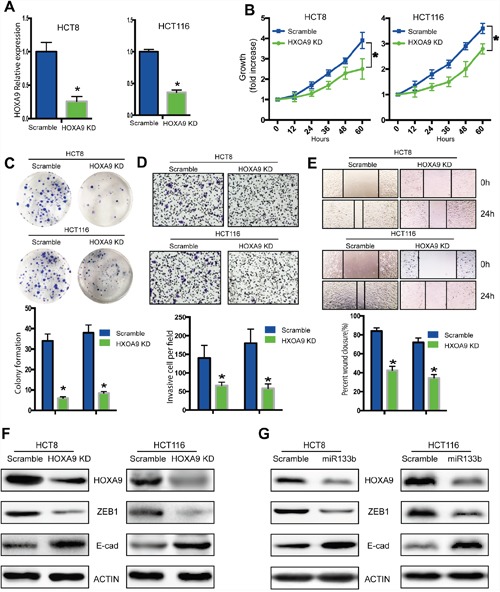
Knockdown of HOXA9 suppresses CRC tumorigenesis and metastasis HCT8 cells were infected with a lentivirus expressing specific shRNA of HOXA9 genes (HOXA9-KD) or shRNA scramble controls (Lv-scramble). **(A)** qPCR analysis of the knockdown effect in HCT8 and HCT116 cells infected with HOXA9-KD or Lv-scramble. **(B)** *in vitro* cell proliferation was detected with CCK8 assay. **(C)** *in vitro* cell proliferation was detected with colony formation assay. **(D, E)** *In vitro* cell migration was analyzed with a gel invasion assay **(D)** and a wound-healing assay **(E)**. **(F)** Knock down of HOXA9 decreased ZEB1 while increased E-cadherin expression. **(G)** Overexpression of miR133-b decreased HOXA9 and ZEB1, while increased E-cadherin expression. *P < 0.05, versus scramble control.

We showed that HOXA9 knockdown significantly decreased the proliferation and colony formation of HCT8 and HCT116 cells (Figures 5A-5C). Knockdown of HOXA9 also declined migration and invasion of CRC cells (Figure 5D, 5E), indicating that HOXA9 mediated the supportive effects on CRC migration and invasion.

### MiR-133b decreased ZEB1 expression by targeting HOXA9 in CRC cells

To illustrate the mechanism of metastasis for miR-133b and HOXA9, we checked the expression of several metastatic markers. We found that knockdown of HOXA9 significantly downregulated ZEB1 expression and upregulated E-cadherin expression in CRC cells (Figure 5F). Remarkably, ZEB1 expression decreased and E-cadherin expression increased following HOXA9 downregulation in miR-133b mimic-transfected cells (Figure 5G). These results suggested that miR-133b might suppress ZEB1 and E-cadherin expression by targeting HOXA9 to suppress CRC cell migration and invasion.

### Rescue experiments further proves the miR-133b - HOXA9 /ZEB1 pathway to reduce tumor metastasis

**Figure 6:**
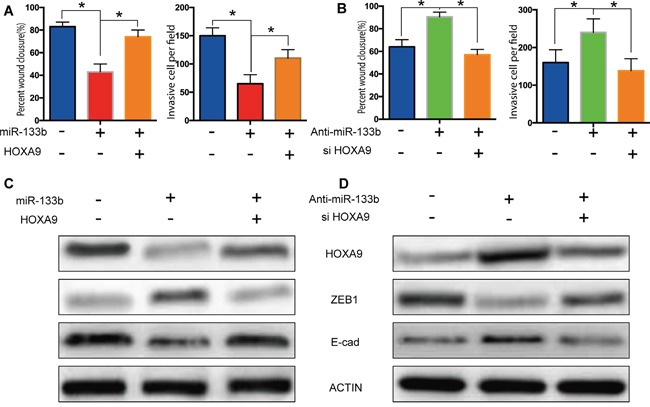
Rescue experiments validated the miR-133b - HOXA9 /ZEB1 pathway **(A)** Restoration of HOXA9 expression in cells expressing miR-133b mimic blocked the miR-133b-induced suppression of migration and invasion. **(B)** Knockdown of HOXA9 abolished migration and invasion elevation in miR-133b inhibited cells. **(C, D)** Re-expression of HOXA9 in cells expressing miR-133b reversed ZEB1 and E-cadherin expression levels alteration induced by miR-133b and knockdown of HOXA9 after miR-133b inhibitor transfection also abrogated protein change of ZEB1 **(C)** and E-cadherin **(D)**.

The restoration of HOXA9 expression in cells expressing miR-133b mimic blocked the miR-133b-induced suppression of migration and invasion (Figure 6A) and knockdown of HOXA9 abolished migration and invasion elevation in miR-133b inhibited cells (Figure 6B), indicating that HOXA9 mediated the suppressive effects of miR-133b on CRC migration and invasion. Remarkably, re-expression of HOXA9 in cells expressing miR-133b reversed ZEB1 and E-cadherin expression levels alteration induced by miR-133b and knockdown of HOXA9 after miR-133b inhibitor transfection also abrogated protein change of ZEB1 and E-cadherin (Figure 6C, 6D). These results suggested that miR-133b might regulate ZEB1 and E-cadherin expression by targeting HOXA9.

### Prognostic significance of miR-133b and HOXA9 in CRC patients

**Figure 7:**
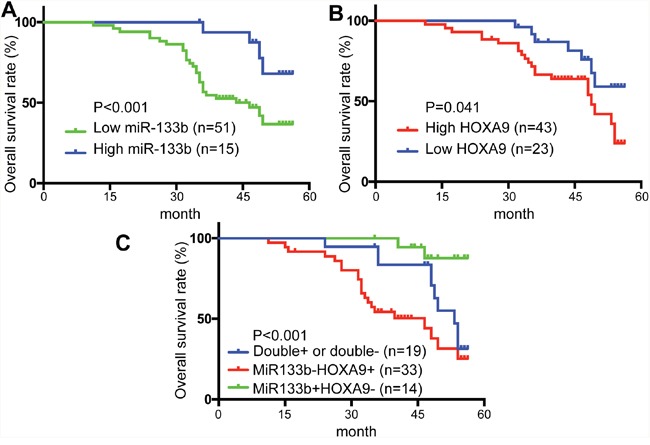
Low level of miR-133b and high level of HOXA9 predicted poor prognosis in colorectal cancer patients **(A)** overall survival (OS) rates of miR-133b in 66 patients by Kaplan–Meier analysis with log-rank test (**P < 0.001). **(B)** overall survival (OS) rates of HOXA9 in 66 patients by Kaplan–Meier analysis with log-rank test (*P = 0.041). **(C)** Overall survival (OS) rates of three groups based on the expression of miR-133b and HOXA9: miR-133b+/HOXA9- (14 specimens); miR-133b+/HOXA9+, or miR-133b-/HOXA9- (19 specimens); miR-133b-/HOXA9+ (33 specimens).

To further evaluate the clinical significance of the miR-133b/HOXA9 axis in CRC, we determined miR-133b and HOXA9 expression levels in 66 CRC patients. CRC patients with higher miR-133b expression levels had better overall survival than the group with lower miR-133b expression levels (Figure 7A), while HOXA9 showed the opposite that high expression predicts worse outcome (Figure 7B). Furthermore, with regard to correspondent expression of miR-133b and HOXA9, we divided the specimens into 3 groups: group 1, tumors exhibiting higher expression of miR-133b and lower HOXA9 (miR-133b+/HOXA9-, 14 specimens); group 2, tumors with both higher or both lower expression of miR-133b and HOXA9 (miR-133b+/HOXA9+, or miR-133b-/HOXA9-, 19 specimens); and group 3, tumors with lower expression of miR-133b and higher HOXA9 (miR-133b-/HOXA9+, 33 specimens). Notably, there was a trend toward a better OS in the patient group with miR-133b+ and HOXA9- than that in the patient group with miR-133b- and HOXA9+ tumors (P<0.001) (Figure 7C). Univariate analyses using the Cox hazard regression model identified low miR-133b expression, high HOXA9 expression, AJCC stage, differentiation and metastasis as prognostic indicators of overall survival for CRC patients (Table 1). Multivariate analysis further demonstrated that, like AJCC stage, differentiation and metastasis, low miR-133b expression was an independent and significant risk factor of overall survival for CRC patients (hazard ratio, 2.977; 95% CI, 1.622-5.283; P < 0.001; Table 2). However, HOXA9 didn't show significant prognostic power in multivariate analysis, which may be due to the small size of our samples. These results revealed a significant contribution of higher miR-133b expression to better CRC patient outcomes and indicated that miR-133b was an independent and significant prognostic factor for CRC patients.

**Table 1:** Univariate analyses of factors associated with overall survial rate in CRC patients

Factors	OS relative risk	95% CI	P value
Gender (male vs. female)	1.224	0.785-2.673	0.387
Age (> 60 vs. <=60)	1.432	0.824-3.321	0.149
Tumor size (>5cm vs. <=5cm)	1.893	0.573-4.328	0.532
Tumor stage (III+IV vs. I+II)	3.345	1.435-6.536	0.002
Tumor location (left vs. right)	0.892	0.346-1.573	0.674
Differentiation (low vs. high)	2.573	1.323-5.423	0.015
Metastasis	3.675	2.035-5.269	0.000
MiR133b expression (low vs. high)	2.743	1.047-5.013	0.000
HOXA9 expression (high vs. low)	1.652	1.015-4.360	0.036

Univariate analysis was performed by the Cox proportional hazards regression model. CI: confidential interval; OS:overall survival.

**Table 2:** Multivariate analyses of factors associated with overall survival rate in CRC patients

Variables	HR	95% CI	P value
Tumor stage (III+IV vs. I+II)	4.263	2.367-6.793	0.000
Differentiation (low vs. high)	1.257	1.029-4.269	0.042
Metastasis	3.922	1.273-6.672	0.000
MiR133b expression (low vs. high)	2.977	1.622-5.283	0.000

Cox multivariate proportional hazard regression was adopted in multivariate analyses. HR: hazard ratio; CI: con dential interval.

## DISCUSSION

Invasion and metastasis are symbols of cancer [18] and critical processes in cancer development. Mounting evidence intimates that the anomalous expression of microRNAs (miRNAs) contributed to tumor evolution [19]; however, the capacity of miRNAs in human CRC remains widely undefined. In this study, we announced that miR-133b is significantly downregulated in CRC samples and cell lines compared to normal controls. We further found that decreased expression of miR-133b in CRC is crucial in the achievement of an advancing and poor prognostic phenotype. Besides, externally induced expression of miR-133b significantly hindered CRC cell proliferation and invasion *in vitro* and *in vivo*. Mechanistically, we distinguished HOXA9 as a direct and practical target of miR-133b. The levels of miR-133b were inversely connected with the expression of HOXA9 in the CRC tissues. We also studied the function of the HOXA9 itself in CRC and proved that the knockdown of this target led to significant repression of tumor cell proliferation and migration. Our findings revealed the crucial roles of miR-133b and its target-HOXA9, in the administration of CRC progress and implemented new latent candidates for CRC therapy.

It is recognized that miR-133b was usually downregulated in various types of human cancer, such as esophageal squamous cell carcinoma [20], glioma [21], bladder cancer [22], non-small cell lung cancer [23], prostate cancer [24, 25], gastric cancer [26] and even colon cancer [27]. However, little is known about the role of miR-133b in mediating the proliferation and invasion of CRC and the underlying mechanism.

As noncoding RNAs, miRNAs execute their capacities by targeting protein-coding genes and binding to the seed sequences in 3′-UTR. HOXA9 was reported to be most commonly altered in solid tumors. However, HOXA9 was significantly downregulated in breast cancers relative to normal breast tissues [28, 29]. HOXA9 inhibits migration of lung cancer cells and its hypermethylation is associated with recurrence in non-small cell lung cancer [30]. These results demonstrated that HOXA9 could be a tumor suppressor in some of the cancer. On the other hand, HOXA9 promotes homotypic and heterotypic cell interactions that facilitate ovarian cancer dissemination via its induction of P-cadherin [31]. HOXA9 are aberrantly expressed during prostate carcinogenesis [32]. High expression levels of HOXA9 were also predictive of shorter survival in glioma patient samples [33]. Overexpression of HOXA9 was seen in epithelial ovarian cancers [34]. It was shown that HOXA9 was independent indicators of prognosis in non-muscle invasive bladder cancers [35]. These results revealed the oncogene side of HOXA9. Until now, the roles of HOXA9 in CRC development and metastasis have been unexplored; here, we proclaimed its involvement in CRC progress for the first time.

HOXA9 was upregulated in CRC samples and positively correlated with tumor cell invasion and metastasis. The overall survival of CRC patients was better in patients with Low HOXA9 expression than in patients with high HOXA9 expression. These results demonstrated that HOXA9 played an important role in CRC metastasis. Moreover, HOXA9 expression was inversely associated with miR-133b expression in CRC samples, which suggested that HOXA9 upregulation in CRC might be caused by miR-133b downregulation. Besides, we demonstrated that HOXA9 is essential for the expression of the zinc finger protein ZEB1, a master regulator of cancer metastasis [36, 37]. We also found that miR-133b decreased ZEB1 expression by downregulating HOXA9 expression in CRC cells. The expression of E-cadherin, a downstream effector of ZEB1 [38], was upregulated by miR-133b and suppressed by HOXA9. Therefore, miR-133b inhibited CRC cell migration and invasion may by downregulating HOXA9 expression and inactivating the HOXA9/ZEB1 signaling pathway.

In conclusion, we newly identified miR-133b/HOXA9/ZEB1 as an important signaling pathway that governed CRC metastasis. MiR-133b and HOXA9 might be useful indicators for CRC patient outcomes, and the miR-133b/HOXA9/ZEB1 pathway might be a promising therapeutic target for CRC treatment.

## MATERIALS AND METHODS

### Cell culture and preparation of clinical samples

Two human CRC cell lines (HCT8, HCT116) were obtained from the American Type Culture Collection (ATCC; Manassas, VA, USA) and were cultured in DMEM (Invitrogen, CA, USA) containing 10% fetal bovine serum (FBS) at 5% CO_2_ Humidity and 37°C. The normal colon epithelial cell line NCM460 was grown in DMEM supplemented with 10% FBS. 66 CRC tissue samples and matched adjacent normal tissues, including 10 liver metastatic patients’ metastatic sample, were obtained from The Fifth Affiliated Hospital of Sun Yat-Sen University, and frozen in liquid nitrogen and stored at −80°C until use. For each CRC patient, the following demographic and clinicopathological information was gathered: age, gender, tumour size, tumour location, histological differentiation, TNM stage and follow-up time after surgery. This study was approved by the institutional ethical review boards of The Fifth Affiliated Hospital of Sun Yat-Sen University, and written informed consent was obtained from all patients.

### RNA extraction from cells and tissues

Total RNA was extracted from tissues and cells using Trizol (Takara, Japan). The concentration and purity of RNA were determined using the NanoDrop® ND-2000 spectrophotometer (NanoDrop Technologies, USA).

### Microarray and qRT-PCR analysis

MiRNA expression profiles of the CRC primary samples and metastatic hepatic samples were evaluated with a bead-based miRNA microarray (Human v2 MicroRNA Expression Profiling Kit, Illumina, USA) containing 1145 human precursor and mature miRNA oligonucleotide probes. Illumina BeadStudio version 3.1 (www.Illumina.com) was used to process signal intensity values from the scans and the background was subtracted. Eisen CLUSTER and TREEVIEW programs were used for hierarchical clustering and visualization of the data. Before applying the clustering algorithm, we log transformed the fluorescence ratio for each expression and then average centered the data for all samples.

For the analysis of mature miRNA, quantitative PCR (qRT-PCR) was performed using the miScript PCR System (Qiagen, Hilden, Germany) according to the manufacturer's instructions. Relative expression was calculated using the comparative CT method and normalized to the expression of U6 small nuclear RNA. The following primers were used: miR- 133b, forward: 5′-GAACCAAGCCGCCCGAGA-3′ and reverse: 5′-CCGCCCTGCTGTGCTGGT-3′; U6, forward: 5′-CTCGCTTCGGCAGCACA-3′ and reverse: 5′-AACGCTTCACGAATTTGCGT-3′. While the primers for human HOXA9 were as follow, forward: 5′-GTGGTTCTCCTCCAGTTGATAG-3′, reverse: 5′-AGTTGGCTGCTGGGTTATT-3′. β-actin was used as an internal control.

Changes in the expression were calculated using the 2^−ΔΔCt^ or 2^−ΔCt^ method.

### Expression data sets

CRC patients from The Cancer Genome Atlas (TCGA, https://tcga-data.nci.nih.gov/tcga/) database (hereinafter referred to as the TCGA cohort) were enrolled in this study. Among them, 231 patients have miR-133b expression data available and 278 patients have HOXA9 expression data available. BioGPS database (biogps.org) could be searched for genes, and each gene's full expression profile is displayed as a bar chart.

### Oligonucleotide and plasmid transfection

MiR-133b mimic, inhibitor and negative control (miR-NC) were designed and synthesized by RiboBio (Guangzhou, China). Small interfering RNAs (siRNAs) targeting *HOXA9* and negative control siRNA were ordered from RiboBio (Guangzhou, China). miRNA probes were purchased from Life Technologies (Shanghai, China). Cells were plated in individual wells of 6-well plates. MiR-133b mimic or miR-NC was transfected into HCT8 and HCT116 cells using Lipofectamine 2000 (invitrogen) according to the manufacturer's protocol. After 48h, the cells were harvested for the assays described below.

### Establishment of HOXA9-Knock down cell lines

pLV- sh-HOXA9 (HOXA9-KD) and empty LV-sh-Scramble Lentivirus (Scramble) was purchased from Genechem Co. Ltd., Shanghai, China. The HCT8 and HCT116 cells were transduced with the lentivirus. Forty-eight hours after infection, 2μg/ml of puromycin was added to the media for 2 weeks to select the cells infected with the lentivirus.

### Cell proliferation assays

Cellular proliferation was measured with the Cell Counting Kit-8 (CCK-8, Dojindo, Japan) assay. Twenty-four hours after transfection, cells were seeded at a density of 5 × 10^3^ cells/well in 96-well culture plates and cultured for 12, 24, 36, 48, or 60h. The cells were then incubated with 10 μl CCK8 for an additional 2h at 37°C. After incubation, the viability of cells was measured at 450nm using a microplate reader (Epoch; BioTek, USA). All experiments were repeated three times.

For the colony formation assay, cells (5 × 10^3^ cells per 10 cm^2^ plate) were seeded in complete medium. The cells were grown for 15 days at 37°C with 5% CO2. Colony formation and growth were visualized with crystal violet staining. The numbers of colonies containing >50 cells were determined, and five fields were counted.

### Transwell invasion assays

Cell invasion assays were performed using Transwell chambers (8 μm, Corning Costar Co., MA, USA). Twenty-four hours after the transfection, 5 × 10^4^ cells in 200 μl serum-free DMEM medium were placed in the 1:10 diluted Matrigel-coated (BD Biosciences, CA, USA) upper chamber. The lower chamber was filled with 500 μl complete DMEM medium. The cells were incubated for 24 h at 37°C and then the cells on the top surface of the membrane were removed by wiping with a cotton swab. The cells that had migrated or invaded from the upper surface to the bottom surface of the filter membrane were stained with 0.5% crystal violet solution and photographed in five present fields per insert. The cells that had migrated to the bottom surface were then eluted with 33% acetic acid and the OD of the positively-stained cells was measured at 570 nm using a microplate reader (BioTek).

### Wound closure assay

Cells were cultured on 6-well plates forming single cell layer, and a straight wound line was made in the middle of the cell layer. After cultured for 24 h, cells that migrated into the wound line were observed. Images of cells along the wound line were taken by phase-contrast microscope (Nikon Digital ECLIPSE C1 system, Nikon Corporation, Japan).

### Luciferase reporter assay

Luciferase constructs were constructed by ligating oligonucleotides containing the wild-type (WT) or mutant putative target site of the HOXA9 3′-UTR into the multi-cloning site of the pGL3-basic vector (Promega, WI, USA). Clones were identified by restriction enzyme digestion (Kpn I and Hind III; Promega) and sequencing. HEK-293T cells were transfected with the reporter vector containing the WT or or mutant target site of the HOXA9 3′-UTR or a control reporter vector with Lipofectamine 2000 and then co-transfected with miRNA miR-133b-mimics or a Scramble control. After 36 h, the luciferase assay was performed using the Dual-Luciferase Reporter Assay System (Promega) in triplicate. Firefly luciferase activity was normalized to that of Renilla luciferase for each sample.

### Western blot

For western blot analysis, proteins (20 μg) were separated by SDS-PAGE and transferred onto polyvinylidene difluoride (PVDF) membranes (Millipore, Billerica, MA, USA). Membranes were blocked in 5% non-fat milk in Tris-buffered saline with 0.05% Tween-20 at room temperature for 2 h and then probed with antibodies against HOXA9, ZEB1 and E-cadherin (1:1000 dilution, Abcam, Cambridge, MA, USA), and probed with goat anti-rabbit secondary antibody conjugated with horseradish peroxidase. Antibodies against β-actin (1:5000 dilution, Sigma, St. Louis, MO, USA) were used as an internal control. The bands were observed with the enhanced chemiluminescence western blotting detection kit (Amersham Life Science Inc., Little Chalfont, UK). Relevant protein expression levels were defined as the ratio of the density of the band for target protein to that of β-actin.

### Tumorigenicity *in vivo*

Male BALB/c nude mice aged 4-6 weeks were obtained from shanghai Laboratory Animal Center of China. For the tumor growth assay, HCT116 and HCT8 cells transfected with miR-133b mimic and miR-NC, were subcutaneously injected into nude mice. Tumor growth was determined by measuring the tumor volume, V= (mm^3^, V=tumor length × tumor width^2^/2) every 3 days using calipers. For portal circulation injections, cells were injected into the spleen followed by removal of the spleen. Animals were excluded from studies if inoculated cells did not arrive in the liver. The animal studies and the experimental protocol were approved by the institutional animal care and use committee of The Fifth Affiliated Hospital of Sun Yat-Sen University. All animal experiments were performed according to the guidelines on the care and use of animals for scientific use.

### Statistical analysis

All data were analyzed using SPSS 21.0 software (SPSS, IL, USA) and expressed as mean ± SD. The mRNA relationship between miR-133b and HOXA9 was analyzed by Pearson's correlation. The two-tailed Student's t-test was used to determine the P-value, and P< 0.005 was considered significant.
